# An image caption model based on attention mechanism and deep reinforcement learning

**DOI:** 10.3389/fnins.2023.1270850

**Published:** 2023-10-05

**Authors:** Tong Bai, Sen Zhou, Yu Pang, Jiasai Luo, Huiqian Wang, Ya Du

**Affiliations:** ^1^School of Optoelectronic Engineering, Chongqing University of Posts and Telecommunications, Chongqing, China; ^2^Chongqing Academy of Metrology and Quality Inspection, Chongqing, China; ^3^Department of Peripheral Vascular (Wound Repair), Chongqing Hospital of Traditional Chinese Medicine, Chongqing, China

**Keywords:** image caption, encoder-decoder architecture, deep neural networks, attention mechanism, deep reinforcement learning

## Abstract

Image caption technology aims to convert visual features of images, extracted by computers, into meaningful semantic information. Therefore, the computers can generate text descriptions that resemble human perception, enabling tasks such as image classification, retrieval, and analysis. In recent years, the performance of image caption has been significantly enhanced with the introduction of encoder-decoder architecture in machine translation and the utilization of deep neural networks. However, several challenges still persist in this domain. Therefore, this paper proposes a novel method to address the issue of visual information loss and non-dynamic adjustment of input images during decoding. We introduce a guided decoding network that establishes a connection between the encoding and decoding parts. Through this connection, encoding information can provide guidance to the decoding process, facilitating automatic adjustment of the decoding information. In addition, Dense Convolutional Network (DenseNet) and Multiple Instance Learning (MIL) are adopted in the image encoder, and Nested Long Short-Term Memory (NLSTM) is utilized as the decoder to enhance the extraction and parsing capability of image information during the encoding and decoding process. In order to further improve the performance of our image caption model, this study incorporates an attention mechanism to focus details and constructs a double-layer decoding structure, which facilitates the enhancement of the model in terms of providing more detailed descriptions and enriched semantic information. Furthermore, the Deep Reinforcement Learning (DRL) method is employed to train the model by directly optimizing the identical set of evaluation indexes, which solves the problem of inconsistent training and evaluation standards. Finally, the model is trained and tested on MS COCO and Flickr 30 k datasets, and the results show that the model has improved compared with commonly used models in the evaluation indicators such as BLEU, METEOR and CIDEr.

## 1. Introduction

In recent years, profound advances have been made in deep learning technology due to the breakthrough in computing power of computers and the surge in data (LeCun et al., 2015). Meanwhile, image caption based on deep learning has also seen significant improvements (Bai and An, 2018; Srivastava and Srivastava, 2018; Liu et al., 2019). Image caption is the intersection of the fields of computer vision and natural language processing, along with its potential value in terms of contributing to visually impaired individuals’ daily life assistance, graphic conversion, automatic title generation and machine intelligence (Hossain et al., 2019; Kang and Hu, 2022). Fundamentally, it involves utilizing techniques grounded in deep learning to interpret a given image and automatically generate descriptive text as if the machine is looking at an image and speaking. Despite its intuitive nature for humans, this process is highly challenging for machines, requiring the accurate interpretation of image content, object relationships and the synthesis of appropriate language. As such, significant research efforts are still required to achieve reliable and effective image caption models that match human-level performance (Anderson et al., 2016; Bernardi et al., 2016).

The advancement of image caption technology is of profound importance in terms of both research and practical application. Its significance is particularly evident in the following areas: firstly, in the field of visual assistance systems, image caption can play a vital role in helping the visually impaired access crucial visual information (Jing et al., 2020; Bhalekar and Bedekar, 2022). By expressing image content comprehensively and concretely, this technology can reduce the obstacles that the visually impaired face in their learning and daily life. Secondly, due to the widespread deployment of cameras and the increasing amount of monitoring data being acquired, the workloads of surveillance personnel have become overwhelming. A system based on image caption can provide summarized information of the monitoring data leading to more efficient work processes (Nivedita et al., 2021). Overall, with the continuous development and maturity of deep learning theory, image caption technology will undoubtedly have an increasingly significant impact on people’s lifestyles, advancing progress across society and industry (Amritkar and Jabade, 2018; Kinghorn et al., 2018).

Image caption has broad application prospects, and more and more researchers begin to study this challenging task. Before the introduction of encoder-decoder architecture, two primary approaches had emerged in the early stages, template-based method and search-based method. The template-based approach generates the final caption from a pre-set sentence template. Farhadi et al. (2010) use detectors to detect objects to form descriptions of images based on language templates. Other researchers use independent corpus construction and more effective semantic analysis models to describe the images. Elliott and de Vries (2015) express target objects in images by means of visual dependency representation, selects the target objects corresponding to the most appropriate features, and fills them in the template. After continuous improvement of the template-based method, although the main object of the image can be recognized accurately, the generated sentences are monotonous and lack some semantic information. The search-based method involves using similarity algorithms to compute the similarity between extracted features and the images stored in a constructed image library, to find out the images in line with the algorithm, and these images have been matched with the corresponding sentence descriptions in advance, which can be fine-tuned for appropriate output. Verma et al. (2013) adopt traditional image feature extraction methods to compare the extracted image features with those in the database, so as to determine the maximum joint probability output in the description tuple. Li and Jin (2016) introduce the reordering mechanism which greatly improves the model performance. The search-based method relies heavily on the constructed search image library, and the results have great uncertainty and poor robustness.

The image caption model based on encoder-decoder architecture is derived from the machine translation model (Cho et al., 2014). The encoder-decoder architecture can directly realize the mapping between the images and the descriptions by learning. And the deep neural network model can learn these mappings from a large amount of data to generate a more accurate descriptions, which makes this method have greater improvement in performance compared with the previous methods. The Multimodal Recurrent Neural Network (M-RNN) model is proposed in Mao et al. (2014), stands out as a pioneering approach utilizing an encoder-decoder architecture, effectively bridging the gap between image and text features through modal fusion. The Neural Image Caption (NIC) model proposed in Vinyals et al. (2015) adopt Long Short-Term Memory (LSTM) to replace RNN, which effectively improves performance and is also the baseline model for many subsequent methods. Deng et al. (2020) introduce an adaptive attention model with a visual sentinel, and introduces the Dense Convolutional Network (DenseNet) to extract the global features of the image in the encoding phase, which significantly improves the quality of image caption generation. Fei (2021) propose a memory-augmented method, which extends an existing image caption model by incorporating extra explicit knowledge from a memory bank, and the experiments demonstrate that this method holds the capability for efficiently adapting to larger training datasets. In Shakarami and Tarrah (2020), an efficient image caption method using machine learning and deep learning is proposed. The experimental results demonstrate the superiority of the offered method compared to existing methods by improving the accuracy. Huang et al. (2019) propose an Attention on Attention (AoA) network for both the encoder and the decoder of the image caption model, which extends the conventional attention mechanisms to determine the relevance between attention results and queries. Krause et al. (2017) use faster-RCNN to acquire regional features and combine them, and then uses multi-layer recurrent neural networks to get the image caption. There are several other improvements (Yang et al., 2019; Liu et al., 2020; Parikh et al., 2020; Singh et al., 2021) that are based on this encoder-decoder architecture. This kind of method is characterized by its flexibility and strong generalization ability. At present, most improvements are based on encoder-decoder architecture.

With the development of technology, the performance of image caption has been made substantial advancements compared with traditional methods (Liu et al., 2020). However, there are several challenges persist, including shortcomings in the encoding and decoding processes, loss of visual information during decoding, insufficient attention to detail information, and discrepancies between training objectives and evaluation indicators. To address these issues, this paper studies and optimizes the image caption model with encoder-decoder architecture. The structure of the paper is arranged as follows: section 2 puts forward the image caption model based on guided decoding and feature fusion. Section 3 further improves the performance of the image caption model. Section 4 provides the experimental process and result analysis. Finally, the conclusion of our image caption model is in section 5.

## 2. Image caption model based on guided decoding and feature fusion

In order to solve the problems in image caption technology, this paper proposes an image caption model based on guided decoding and feature fusion. Based on the encoder-decoder architecture, DenseNet model is used to encode image features, and the Multiple Instance Learning (MIL) method is used to extract the image visual information. The two parts together constitute the encoding process of image visual information, and the guided decoding module is adopted to dynamically adjust the input image visual information during the decoding process. The decoder uses a Nested Long Short-Term Memory (NLSTM) network, which can learn more hidden information by increasing the depth of the network model.

### 2.1. Encoder design based on feature fusion

**FIGURE 1:**
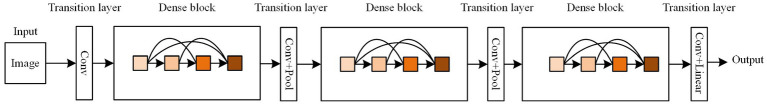
DenseNet model.

Convolutional Neural Network (CNN) is a crucial model for processing visual image problems and have significantly improved with each architecture iteration. Typically, lower-level features are utilized to distinguish between various classes of basic contour information, while higher-level features are more abstract and effectively differentiate between different varieties of semantic information for the same target. From this perspective, the deeper the layers of the network model, the richer the information extracted. However, the consequent problem is that the increase in model depth causes the gradient to diminish until it disappears during the transfer process. The problem of gradient disappearance can be solved to some extent by using the Batch Normalization (BN) method (Bjorck et al., 2018). Residual Network (ResNet) and highway network also address the problem of gradient disappearance and model degradation by using bypass settings and gating units (Shaked and Wolf, 2017). Nevertheless, these models are prone to excessive parameters and depth redundancy. In image caption tasks, where image scenes are rich, it is necessary not only to identify targets but also to be able to abstractly describe the interconnections between targets, so fusing the base feature map with higher-level feature maps is a good way to handle this problem. In this paper, we employ the DenseNet model for image feature extraction, which is based on the architecture as illustrated in Figure 1. The fundamental concept of DenseNet resides in establishing connections between varied depth feature maps, enabling the utilization of both high-level and low-level features to their fullest potential.

DenseNet has been identified to improve feature multiplexing by means of bypass and this not only deepens the network’s layer depth, but also amplifies image information availability. Furthermore, it mitigates problems related to gradient disappearance and model degradation while also keeping the number of parameters less than those of deep neural networks such as ResNet. Meanwhile, with the increase in layer depth, optimization of the network does not become more convoluted. The model’s accuracy increases proportionally with an increase in parameters, devoid of overfitting occurrences.

For the image caption tasks, the object, attribute and relation detector are trained separately by independent hand-labeled training data. We train our image caption models on datasets that contain multiple images and descriptive sentences corresponding to each image. Different from the tasks of image classification and object detection, in the task of image caption, there are not only nouns, but also verbs, adjectives, adverbs and other parts of speech in the description generated by an image. Therefore, in order to describe the needs of the tasks, it is necessary to construct a word set *D* composed of 1,200 common words, which basically contains more than 95% of the words that need to be used in the training set, and the remaining words are treated as non-essential words.

Then, we need to extract the corresponding word from the image through the constructed word set. Because the datasets used in this paper did not define and label corresponding words with corresponding bounding boxes, at the same time, the parts of speech are not even marked, typical supervised learning methods are not suitable for this task. Certainly, while image classification can provide corresponding words for a whole image, many words or semantics are only applicable to the subregions of the image. Such generic classifications often fail to enhance model performance. Therefore, this study applies the MIL method to tackle tasks with one-to-many relationships (Dietterich et al., 1997).

In the image caption tasks, each image corresponds to a packet. For each word *w* in the word set *D*, the packets are divided into positive packets and negative packets according to different image areas, thus forming the input set of the whole MIL model. The classification method is as follows: if the word *w* in the word set *D* appears in the corresponding description sentence of an image *I*, then the packet is marked as a positive packet; if the word in the word set has no corresponding word in the description sentence, the packet is marked as a negative packet. The training set is represented in formula (1).


(1) $$\left\{\left(x_{1},y_{1}\right),\left(x_{2},y_{2}\right),\ldots ,\left(x_{l},y_{l}\right)\right\}$$

For the input packet in the training set $x_{i}$, when $y_{i}=1$, it is the positive packet, and when $y_{i}=-1$, it is the negative packet. Using the MIL model, the probability $P_{w}$ that each packet $b_{i}$ contains the word *w* in the word set *D* is calculated by the following formula:


(2) $$P_{w}=1-\prod_{j\in b_{i}}\left(1-x_{\mathrm{ij}}^{w}\right)$$

Where $x_{\mathrm{ij}}^{w}$ represents the probability that a particular region *j* in an image *i* corresponds to the word *w* in the word set. Since it is image information, the Visual Geometry Group Network (VGGNet) model is used here for calculation. VGG16 model has a total of 16 layers, including 5 convolutional layers, each convolutional layer is followed by a pooling layer, generally using the maximum pooling method. After the convolutional layers, there are 3 fully connected layers, and finally the SoftMax layer is used for classification. The input of the network model is a 224*224 RGB image. The specific calculation process of $x_{\mathrm{ij}}^{w}$ is to adopt a fully connected layer with a sigmoid nonlinear activation function, and the formula is as follows.


(3) $$x_{\mathrm{ij}}^{w}=\frac{1}{1+\exp\left(-W_{w}^{t}\theta \left(b_{\mathrm{ij}}\right)+b_{w}\right)}$$

Where $\theta \left(b_{\mathrm{ij}}\right)$ represents the features of region *j* in the image *i* extracted by the seventh fully connected layer in the model, *W_w_* and *b_w_*, respectively, represent the weight and bias of the word *w*, which can be obtained by learning in model training.

After the operation of the model, a spatial feature map of the image will be obtained in the last fully connected layer, which is corresponding to the position of the input image, that is, the features of different regions in the image. The visual text information of the images in datasets is generated by the MIL model. Generally, the top 10 words with the highest probability after being processed by the MIL model are selected.

**FIGURE 2:**
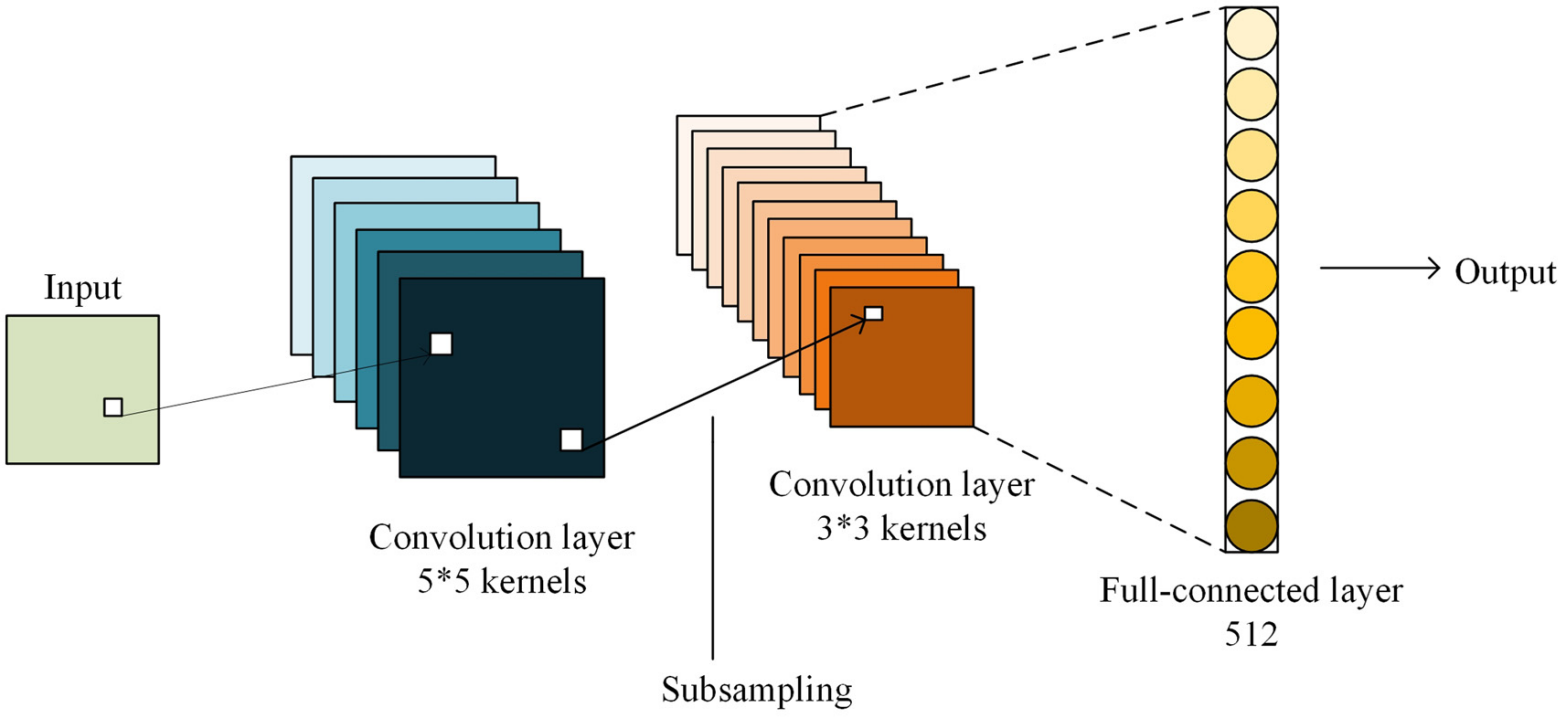
The model structure of guided decoding network.

In this paper, the image feature extraction module and visual information extraction module will be fused by guiding the decoding module to provide a basis for the subsequent decoding process. In the NIC model of image caption, visual information is only input to the decoder at the beginning of decoding, and the strength of its information features will gradually diminish during the decoding process. The ideal decoder should be able to balance the two-input information of image vision and description, so as to avoid the reduction of decoding accuracy because one information dominates the decoding. Therefore, a CNN model for guided decoding is constructed in this paper. By inputting the learned features into the network for modeling, the modeled guidance vector is sent into each time sequence of the decoder, and at the same time, it can accept the error signal feedback from each time sequence of the decoder and make corresponding adjustments. The introduction of the model structure can realize the complete end-to-end training process. The guided decoding network is a deep neural network composed of two convolutional layers and one fully connected layer, represented by CNN-g. Its model structure is shown in Figure 2.

### 2.2. Decoder design based on NLSTM model

Text information is a critical component of training datasets and plays a vital role in the effectiveness of decoding. To ensure optimal feature extraction and expression, it is necessary to structure raw unstructured text data using a text representation model. This allows for efficient participation in the decoder’s training process.

**FIGURE 3:**
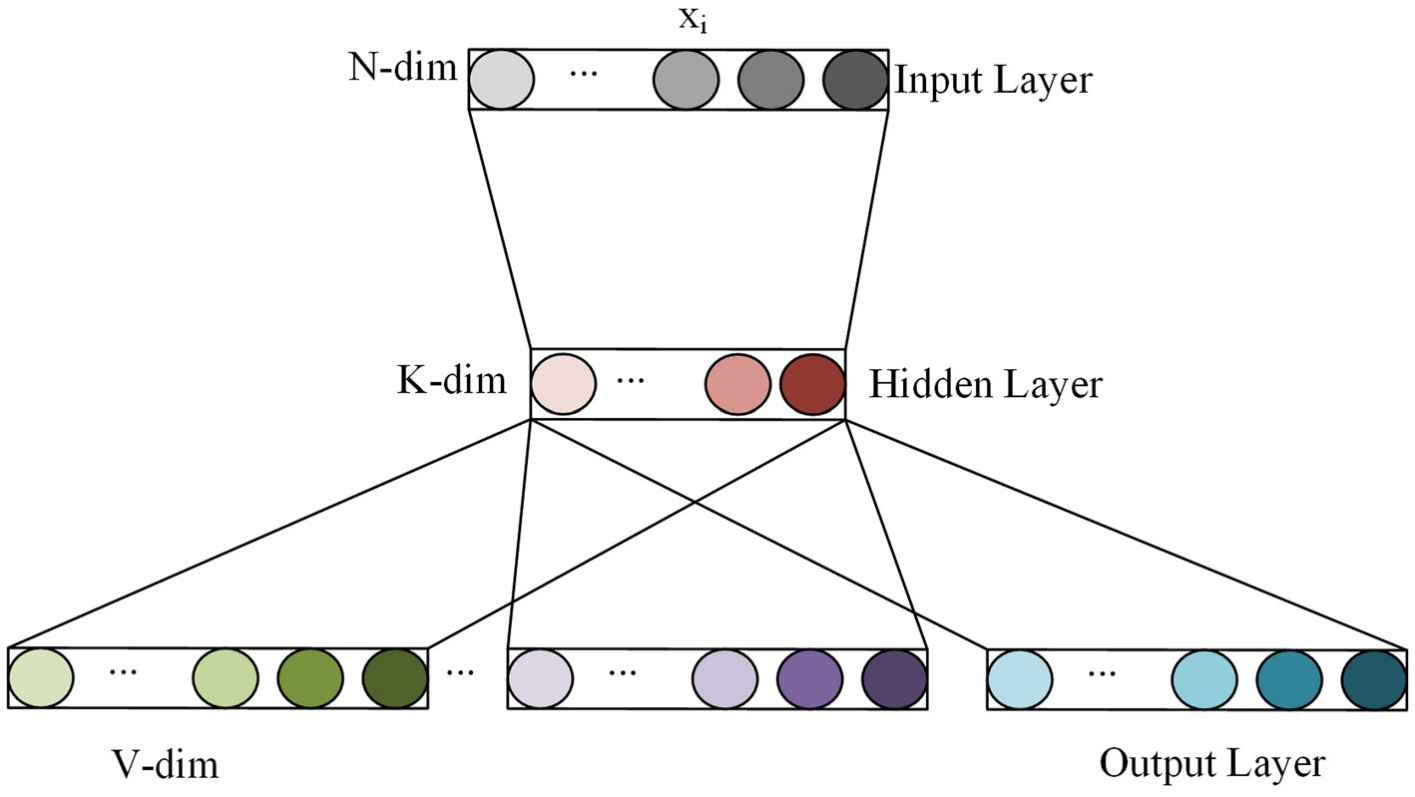
The simplified structure of skip-gram.

Word to Vector (Word2Vec), a highly effective word embedding model built using shallow neural networks, consists of two main structures: skip-gram and CBOW (Continuous Bag of Words). While skip-gram predicts the probability of generating surrounding words based on the current word, CBOW predicts the generation probability of the current word based on surrounding words. The complexity and variation of the semantic environment in image caption require more precise word embedding inputs. To address this need, this paper adopts the skip-gram model. Skip-gram is a shallow neural network model composed of the input layer, hidden layer and output layer, and its simplified structure is shown in Figure 3. Wherein, each word in the input layer uses one-hot encoding, the size of the training set thesaurus is *N*, and the hidden layer has *K* hidden units. After the training is completed, any word *x_i_* in the thesaurus can be calculated to get the feature vector with this word as the central word.

In the actual model training process, managing the number of output feature vectors can pose a challenge due to the large volume of training data involved. To address this issue, the hierarchical SoftMax method is leveraged in this paper. This method entails constructing a Huffman coded binary tree based on word frequencies, where high-frequency words are placed at the root node to minimize computations. The tree is organized hierarchically from top to bottom, with each node classified by a sigmoid activation function. The sigmoid activation function determines the probability of the left and right branches of the tree, and the goal of model training is to multiply the probability on the passed branches to reach the maximum value.

In the context of processing and predicting sequence data, Recurrent Neural Network (RNN) and Long Short-Term Memory (LSTM) networks are commonly employed. When it comes to image caption tasks, RNN and LSTM serve as decoders. Among them, LSTM has proven effective in addressing the long-term dependence issue. In this paper, an enhanced NLSTM model is utilized as a decoder to decode input image features. Different from the general LSTM model, in NLSTM, the memory function $c_{t}$can be obtained through model training as shown in formula (4).


(4) $$c_{t}=m_{t}\left(f_{t}⊙c_{t-1},i_{t}⊙\text{Tanh}\left(w_{c}x_{t}+u_{c}h_{t-1}\right)\right)$$

Where $m_{t}$ is a state function learned from NLSTM, and represents the state *m* at time *t*. $h_{t}$ and $x_{t}$ are the input and hidden states of the memory function, respectively. $i_{t}$ and $f_{t}$ respectively represent the input gate and forgetting gate. *w_c_* and *u_c_* are learned during training.

In the NLSTM model, the specific calculation method of internal LSTM is obtained by the following formulas:


(5) $$\overset{˜}{i_{t}}=\overset{˜}{\sigma_{i}}\left(\overset{˜}{w_{i}}\overset{˜}{x_{t}}+\overset{˜}{u_{i}}\overset{˜}{h_{t-1}}+\overset{˜}{b_{i}}\right)$$

(6) $$\overset{˜}{f_{t}}=\overset{˜}{\sigma_{f}}\left(\overset{˜}{w_{f}}\overset{˜}{x_{t}}+\overset{˜}{u_{f}}\overset{˜}{h_{t-1}}+\overset{˜}{b_{f}}\right)$$

(7) $${\tilde{o}}_{t}={\tilde{\sigma }}_{o}\left({\tilde{w}}_{o}{\tilde{x}}_{t}+{\tilde{u}}_{o}{\tilde{h}}_{t-1}+{\tilde{b}}_{o}\right)$$

(8) $${\tilde{c}}_{t}={\tilde{f}}_{t}⊙{\tilde{c}}_{t-1}+{\tilde{i}}_{t}⊙\text{Tanh}\left({\tilde{w}}_{c}{\tilde{x}}_{t}+{\tilde{u}}_{c}{\tilde{h}}_{t-1}+{\tilde{b}}_{c}\right)$$

(9) $${\tilde{h}}_{t}={\tilde{o}}_{t}⊙{\tilde{\sigma }}_{h}\left({\tilde{c}}_{t}\right)$$

Where ${\tilde{c}}_{t}$ is the internal memory function, ${\tilde{x}}_{t}$ and ${\tilde{h}}_{t}$ are the input layer and hidden layer states of the memory function, respectively. ${\tilde{i}}_{t}$, ${\tilde{f}}_{t}$, and ${\tilde{o}}_{t}$ respectively represent the input gate, forgetting gate and output gate of the internal LSTM. To achieve the gating effect in the neural network, the sigmoid function $\tilde{\sigma }$ is commonly used as the activation function, and the Tanh function is utilized as the candidate memory function. The parameters $\tilde{w}$, $\tilde{u}$, and $\tilde{b}$ are learned during training.

The memory unit of the external LSTM is updated according to formula (10).


(10) $$c_{t}={\tilde{h}}_{t}$$

The value of $h_{t}$ is then updated through the memory unit $c_{t}$ of the external LSTM as shown in formula (11).


(11) $$h_{t}=o_{t}⊙\text{Tanh}\left(c_{t}\right)$$

**FIGURE 4:**
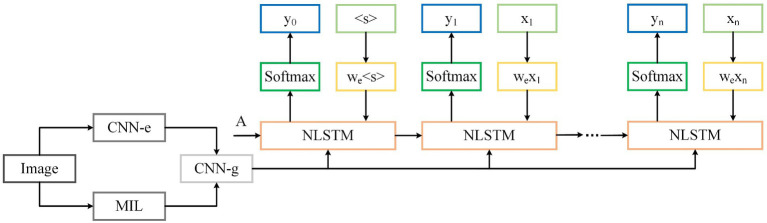
The structure of the image caption model.

NLSTM uses the standard LSTM network as a gating unit to input relevant information into its memory unit, reducing internal memory burden. This enables a more deterministic time hierarchy and better handling of time series problems compared to stacked models. Finally, a SoftMax layer is used in the model to predict the output words obtained by the final model through the probability distribution of words at time *t*. The structure of the image caption model is shown in Figure 4.

In Figure 4, CNN-e represents the DenseNet model used in the coding process, and CNN-g is the guided decoding network. The extracted image fusion features are represented by the formula (12).


(12) $$v=f_{g}\left(A+M\right)$$

Where *A* represents the global image feature, *M* stands for the visual text information learned from multiple instances, and $f_{g}$ represents the model function learned by guiding the decoding model.

The decoded output $y_{t}$ at time *t* is calculated by formula (13).


(13) $$y_{t}=w_{v}v+w_{e}x_{t}$$

## 3. Image caption combining attention mechanism and deep reinforcement learning

In order to further improve the performance of the image caption model, we build a double-layer decoding network by introducing the attention mechanism on the basis of the model proposed above. The output of the first layer and the image features are sent to an attention module to extract important detail features. The output of the module is fused with the output of the first layer as the input of the second layer for the second decoding. Meanwhile, considering the powerful perception and decision abilities of Deep Reinforcement Learning (DRL), this paper constructs a training optimization method based on DRL to improve the overall performance of the model.

### 3.1. Attention mechanism

Although the traditional encoder-decoder based image caption model can describe the content of the image in a short text description, it often ignores some local and detailed information in the image during the description process. However, this information is very important to the richness and accuracy of the description. When the attention mechanism was introduced into the image caption task for the first time, which effectively improved the performance of the NIC model. The attention mechanism is inspired by the human process of observing things, people immediately focus on the important information in an image while paying less attention or ignoring irrelevant information or background information. In deep learning, the formation of attention is basically through the way of masks, that is, important information in the image is distinguished by giving different weights. After continuous training of the model, it can learn which regions are important in the image and form more attention to these regions. There are two main types of attention mechanisms: hard attention and soft attention. Here, we represent the feature vector *v* extracted by the encoder as shown in formula (14).


(14) $$v=\left\{v_{1},v_{2},\ldots v_{k}\right\},v_{i}\in {\mathbb{R}}^{g}$$

The output of the last convolutional layer of the DenseNet is used to represent the features of different positions in the image. At different moments of decoding, the attention weights for different regions of the image can be calculated by formula (15).


(15) $${\hat{v}}_{\mathrm{it}}=f_{\mathrm{att}}\left(v_{i},h_{t-1}\right)$$

Where $h_{t-1}$ represents the state of the hidden layer on the decoder LSTM at time t−1, $f_{\mathrm{att}}$ represents a function that assigns different weights to each region of the image.

The SoftMax function is used to normalize formula (15) so that the weight range is [0,1] and the weighted sum is 1, as shown in formula (16).


(16) $$a_{\mathrm{it}}=f_{\text{soft}\max}\left({\hat{v}}_{\mathrm{it}}\right)$$

Finally, the visual context vectors of different regions of the image are calculated by weight. Its visual context features ${\hat{v}}_{t}$ are expressed as shown in the formula (17).


(17) $${\hat{v}}_{t}=\sum_{i=1}^{k}h_{\mathrm{it}}v_{i}$$

Where $h_{\mathrm{it}}$ is the multivariate two-point distribution of the input vector *v*, $a_{\mathrm{it}}$is the weight of the different regions of the image in the input decoder at time *t*, as shown in formula (18).


(18) $$Ρ\left(h_{\mathrm{it}}|v\right)=a_{\mathrm{it}}$$

**FIGURE 5:**
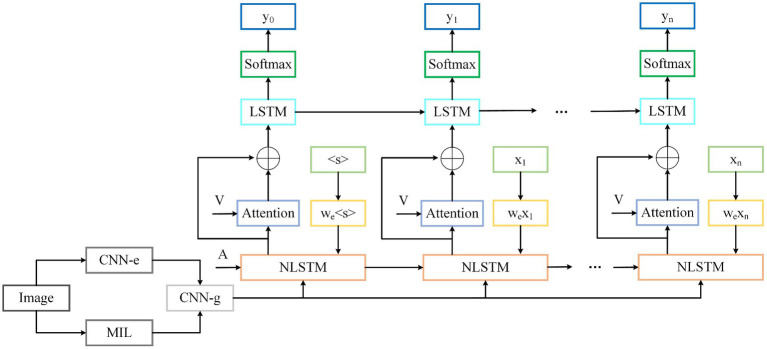
The improved structure of the image caption model.

To obtain local image details during the decoding phase, we propose a double-layer stacked decoding structure, based on the previous model in Figure 4 as the first layer decoding. The new model is depicted in Figure 5. After the output of the first layer decoder and the visual features of the image are calculated by the attention module, they are used as the input of the second layer LSTM decoder by means of residual connection. The introduction of the attention mechanism can effectively improve the performance of the model. The feature vector of the image is represented and calculated by formula (19).


(19) $$v=w^{\mathrm{vi}}f_{\mathrm{cnn}}\left(I\right)$$

Where *I* represents the input original image after preprocessing, $f_{\mathrm{cnn}}$ represents the computational model of DenseNet.

In this model, the last fully connected layer in Figure 4 is removed, and the output of the convolution model is reduced dimensionality by the matrix. The state of the hidden layer of the first layer decoder at time *t* is calculated by formula (20).


(20) $$h_{t}^{1}=f_{\text{nlstm}}\left(x_{t},h_{t-1},v_{g}\right)$$

Where $x_{t}$ represents the input feature vector of word embedding, $h_{t-1}$ represents the hidden layer state at the moment t−1, $v_{g}$ represents the input vector to guide the decoding, and $f_{\text{nlstm}}$ stands for the NLSTM network used by the decoder of the first layer.

In the attention module, the image features and the hidden layer state of the first layer decoder are used as inputs, and unlike the hidden layer state of the t−1 moment used by the soft attention mechanism, the hidden layer state of the *t* moment used here is shown in formula (21).


(21) $${\hat{v}}_{\mathrm{it}}=\text{Tanh}\left(w^{v}v⊕w^{h}h_{t}^{1}\right)$$

Where $w^{v}$ and $w^{h}$ represent the parameter matrix to be learned by the model, ⊕ represents the summation operation of the matrix.

The weight of the attention module is calculated as shown in formula (22).


(22) $$a_{t}=f_{\text{soft}\max}\left(w^{a}{\hat{v}}_{\mathrm{it}}\right)$$

Where $w^{a}$ represents the parameter matrix to be learned by the model, $f_{\text{soft}\max}$ represents the SoftMax operation.

Based on the weight of the attention module, we can get the visual attention features of the image ${\hat{v}}_{t}$, as shown in formula (23).


(23) $${\hat{v}}_{t}=a_{t}v$$

Then, by means of residual connection, the visual attention feature is added and fused with the corresponding subscript element of the hidden layer state $h_{t}$ at *t* moment of the first layer decoder, as shown in formula (24), and it is used as the input of the second layer decoder.


(24) $$x_{t}^{2}={\hat{v}}_{t}⊕h_{t}^{1}$$

LSTM is used as the second layer decoder for the final processing of sequence information. The hidden layer state of the second layer decoder is obtained by formula (25).


(25) $$h_{t}^{2}=f_{\text{lstm}}\left(x_{t}^{2},h_{t-1}^{2}\right)$$

Where $h_{t-1}^{2}$ represents the hidden layer state of the second layer decoder at time *t* − 1, $f_{\text{lstm}}$ represents the model calculation function of the second layer LSTM.

After the second hidden layer state is obtained, an evaluation module is used to predict the possibility of output words, which is mainly composed of linear layer, fully connected layer and SoftMax layer. The linear layer is used for dimensionality reduction of words output by LSTM, and the fully connected layer is used for the upsampling of vectors after dimensionality reduction. Finally, the probability distribution $y_{t}$ of word output is calculated through the SoftMax layer, as shown in formula (26).


(26) $$y_{t}=f_{\text{soft}\max}\left(w^{N}h_{t}^{2}+b^{N}\right)$$

**FIGURE 6:**
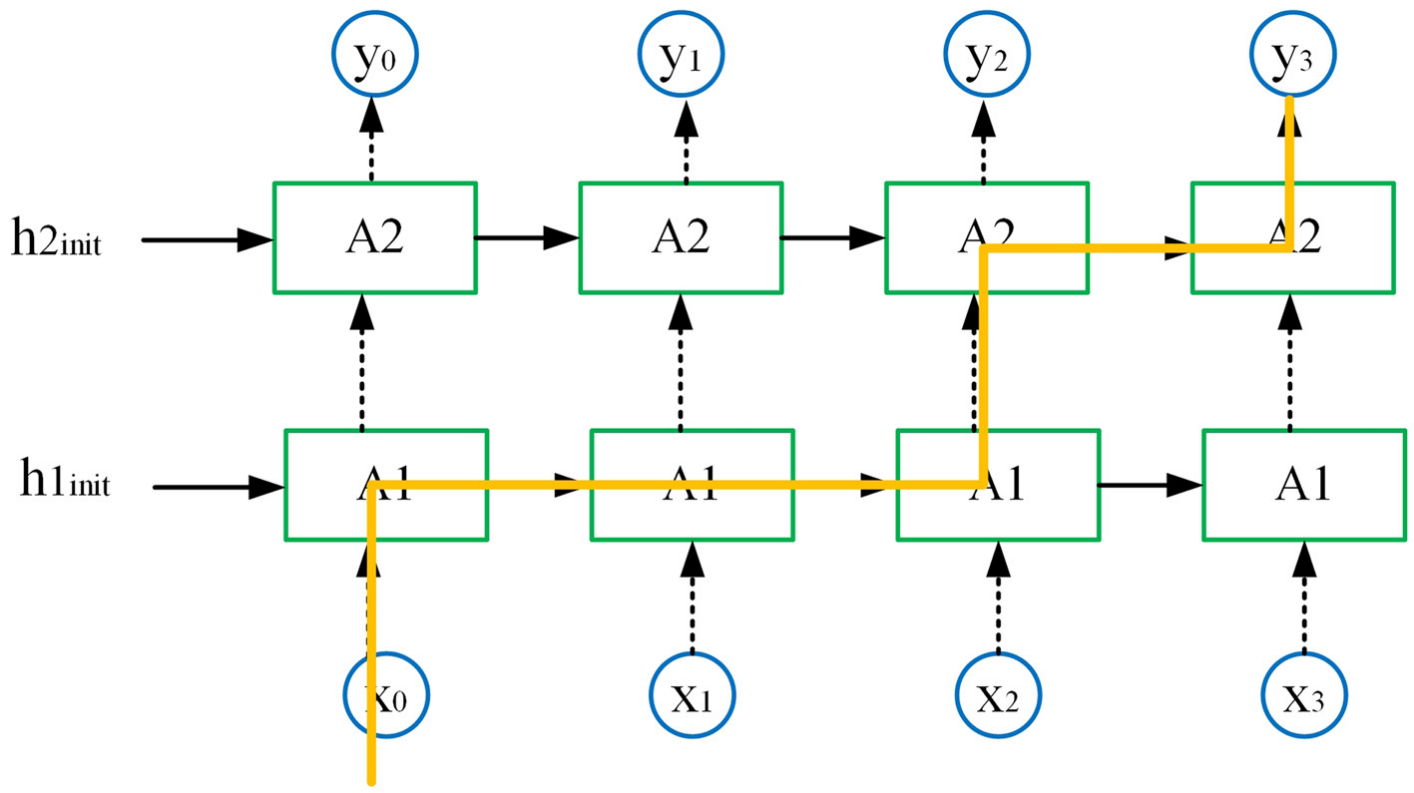
Dropout operation in the double-layer decoding structure.

With the increase of the number of model layers, the expressiveness of the model is also enhanced. However, this also leads to overfitting problems. To address this issue, this paper adopts the dropout method in the double-layer decoding structure that reduces overfitting. The main idea of this method is to deactivate part of the computing units and keep the other part of the computing units working on the data that flows into each unit. Figure 6 illustrates the implementation of dropout operation in the double-layer decoding structure, at time t=0, input $x_{0}$ is passed into the first layer of RNN, and then transmission continues in the first layer until time t=2, during which there is no dropout operation. At time t=2, the dropout operation is performed when the first layer passes to the second layer, which is always coherent in timing. The dropout operation helps greatly in improving the robustness of the model.

### 3.2. Deep reinforcement learning

Reinforcement learning is an artificial intelligence learning method. Different from supervised learning and unsupervised learning, reinforcement learning will only make different rewards or punishments according to the quality of actions. DRL not only has the understanding ability of deep learning, but also makes use of reinforcement learning to make decisions and judgments on the environment, and realizes the response and treatment of complex problems through the end-to-end learning process. The framework of DRL is mainly derived from Markov Decision Process (MDP).

**FIGURE 7:**
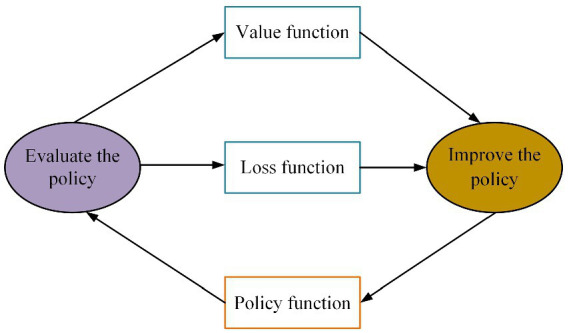
Policy gradient learning method based on actor-critic architecture.

The policy gradient algorithm is a frequently adopted technique for DRL. It offers a direct approach to optimize the expected reward of the policy, without relying on intermediate stages, and enables the determination of an optimal policy within the given policy space. The method utilizes an approximation function to directly optimize the policy and achieve the highest expected total reward. The actor-critic architecture diagram for this algorithm is illustrated in Figure 7, with its policy gradient being expressed through the formula (27).


(27) $$g_{p}=Ε\left(\sum_{t=0}^{\infty }\psi_{t}\nabla_{\theta }\log\pi_{\theta }\left(a_{t}|s_{t}\right)\right)$$

Where $\pi_{\theta }\left(a_{t}|s_{t}\right)$ represents the policy function, which is learned by the neural network in DRL, $\psi_{t}$ represents the evaluation function, which is approximated by a neural network.

The policy function can guide the agent’s actions. The guidance process is calculated according to the probability of taking an action in a certain state, and it is a mapping function from state to action. At the same time, the optimal policy is selected to guide the value function through policy evaluation. The value function is the state value function under the guidance of the policy. The policy function $\theta_{t}$ is updated by formula (28) during the learning process. The value function $w_{t}$ is updated by the formula (29).


(28) $$\theta_{t+1}=\theta_{t}+\mathrm{a\delta}\nabla_{\theta }\log\pi \left(a_{t}|s_{t},\theta_{t}\right)$$

(29) $$w_{t+1}=w_{t}+\beta \delta \nabla_{w}\hat{v}\left(s_{t},w_{t}\right)$$

Where $a_{t}$ and $s_{t}$, respectively, represent the action and state at time *t*.

**FIGURE 8:**
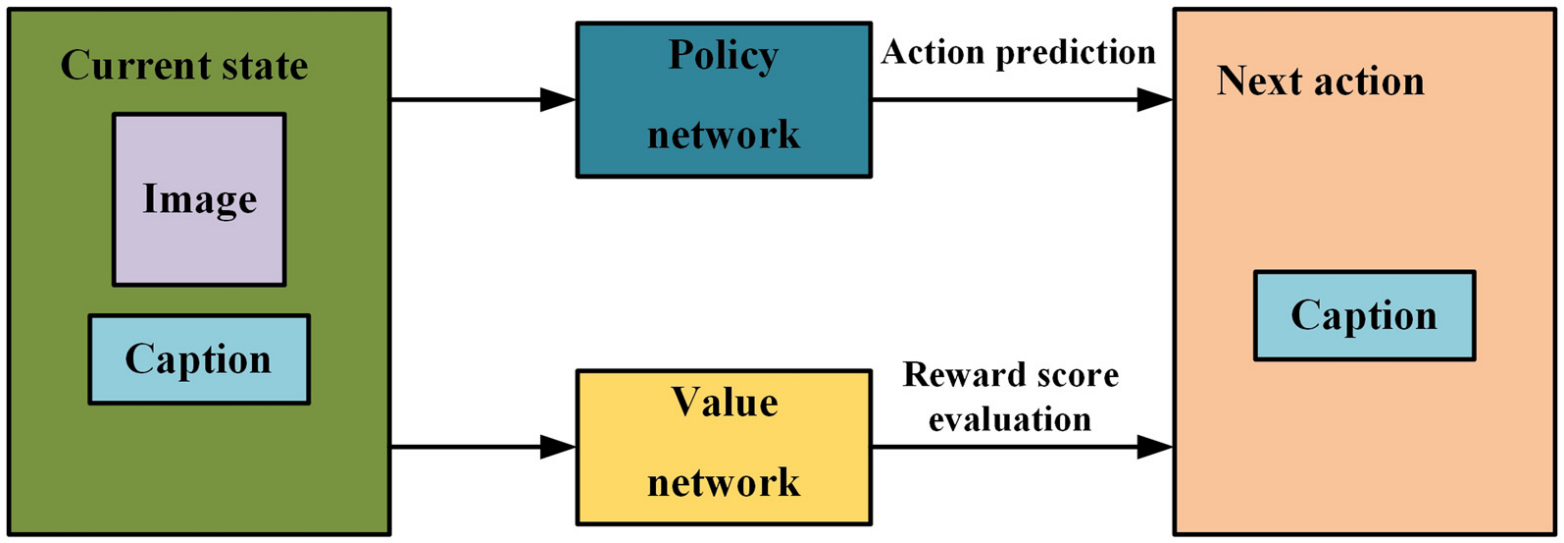
The model structure and prediction process based on DRL.

Considering the powerful perception and decision abilities of DRL, we use it to further optimize our image caption model. And on the basis of the actor-critic structure, two kinds of deep neural networks, policy network and value network, are used to construct models for predicting words that best describe the image in each state. Specifically, the policy network evaluates the confidence of the next predicted word based on the current state, and thus suggests the next possible action to be taken. The value network evaluates the reward scores of the actions predicted by the policy network in the current state, and decides whether to choose the actions given by the policy network according to these reward scores. In other words, the model’s predictions are constantly adjusted according to the actual situation for producing the better image caption. The model structure and prediction process are shown in Figure 8.

The whole process consists of four main elements, including agent, environment, action and goal. In the image caption tasks, the policy network and the value network are the agents and also the main parts of the model. The input image *I* and its description sentence $s_{t}=\left\{x_{1},x_{2},\ldots x_{t}\right\}$ represent the actual environment of the agents. The next predicted word $x_{t+1}$ is the next action, and the thesaurus of all the words in the caption is the space for the actions. Generating the image caption is the goal of this process.

The policy network adopts the encoder-decoder architecture mentioned above in this paper. We use $s_{t}$ to represent the current state, $e=\left\{I,x_{1},x_{2},\ldots x_{t}\right\}$ to represent the environment, and $a_{t}=x_{t+1}$ to represent the next action based on the environment. The visual feature $v_{g}$ of image *I* is extracted by CNN, as shown in formula (30).


(30) $$v_{g}=f_{\mathrm{cnn}}\left(I\right)$$

Using $v_{g}$ as the input of the decoder NLSTM, the action $a_{t}$ at time *t* is predicted according to the hidden layer state $h_{t}$ at time *t* and the input word $x_{t-1}$ at time t−1. Because the decoder adopts a sequential processing mode, the prediction word $x_{t}$ will also be used as the input for time t+1, and the hidden layer state at the next time will also be updated as the input is updated. The formulas are shown as follows.


(31) $$h_{t}=\text{NLSTM}\left(\psi \left(x_{t-1},v_{g}\right),h_{t-1}\right),\;t\in N^{*}$$

(32) $$p_{\varepsilon }\left(a_{t}|s_{t}\right)=\varphi \left(h_{t}\right)$$

Where ψ and φ represent the input and output of the decoder, respectively. $p_{\varepsilon }\left(a_{t}|s_{t}\right)$ represents the possibility of taking action $a_{t}$ in the case of determining state $s_{t}$.

In the value network, the value function $v_{p}$ under the policy *p* is first defined, which represents the prediction of the total reward *r* in the state $s_{t}$, expressed by formula (33).


(33) $$v_{p}\left(s\right)=Ε\left(r|s_{t},a_{t\ldots T}\sim p\right)$$

**FIGURE 9:**
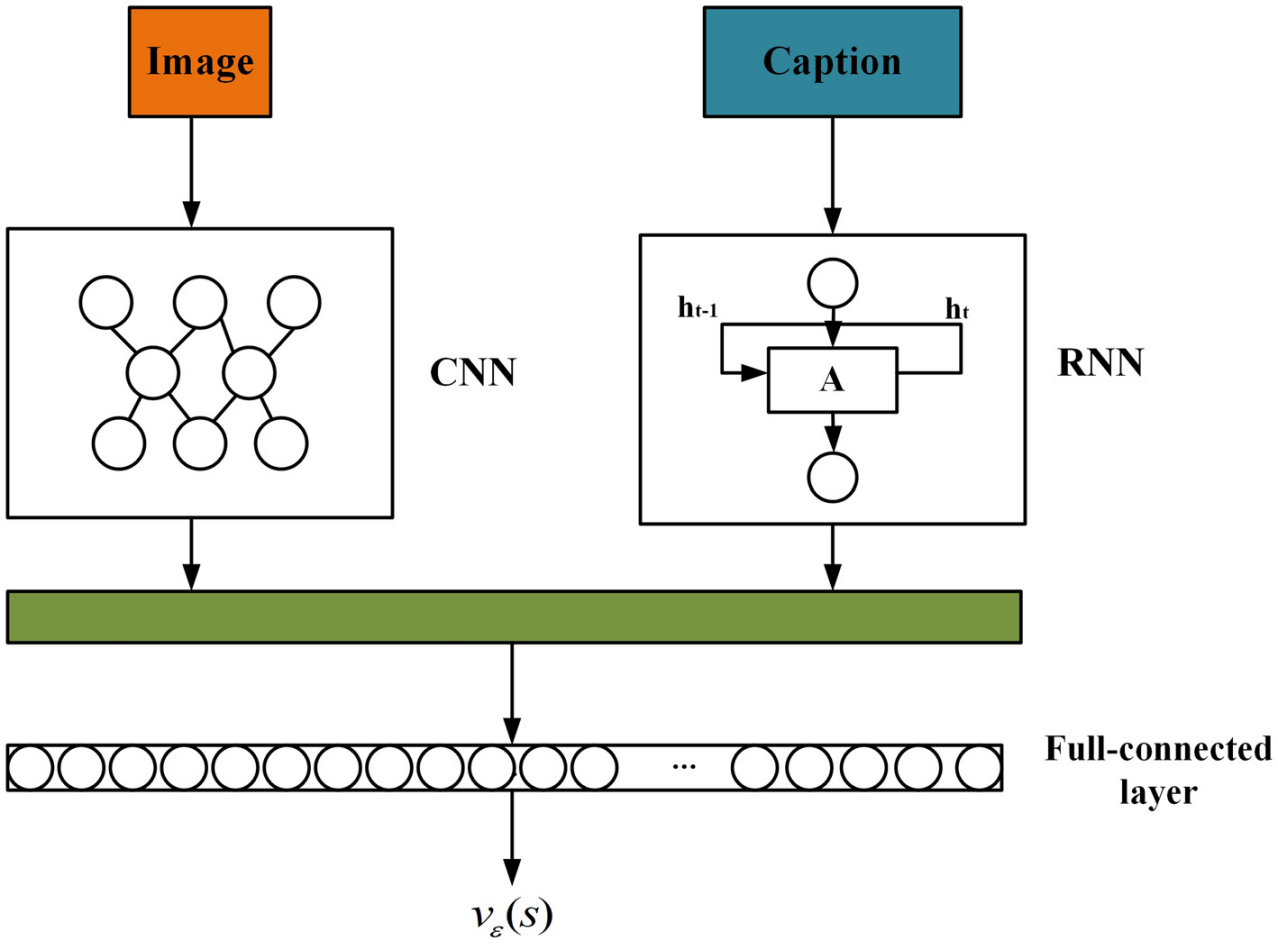
The structure of value network.

In this paper, the output $v_{\varepsilon }\left(s\right)$ of the value network is constructed to fit the value function. The value network is based on the deep neural network, and its structure is shown in Figure 9. It mainly consists of three parts: CNN module, RNN module and fully connected network module. The CNN module is used to extract the visual features of the image, and the Inception-v3 model is selected in this paper. RNN module adopts LSTM structure to extract semantic features of descriptions. The fully connected network module uses the linear regression method to obtain the reward score of the generated semantic descriptions.

In the value network, when the agents complete a goal, the total reward is used to motivate the actions taken. Here, the linear mapping method implemented by the fully connected module maps the image and the corresponding description into a semantic embedding space, to calculate the vector distance between them. The loss function $m_{\text{loss}}$ of this mapping can be expressed by the formula (34).


(34) $$m_{\text{loss}}=\sum_{f_{\mathrm{cnn}}}\sum_{s}\alpha [\max\left(0,h_{T-1}\left(s\right)\cdot f_{m}\left(f_{\mathrm{cnn}}\right)\right)-h_{T}\left(s\right)\cdot f_{m}\left(f_{\mathrm{cnn}}\right)rsqbr$$

Where α is the penalty coefficient with the range of (0,1), $f_{\mathrm{cnn}}$ is the image feature extracted by the DenseNet, and $f_{m}$ is the mapping function.

For a given description sentence *s*, whose embedded characteristics depend on the final state $h_{T}$ of the hidden layer, and the total reward is defined as shown in formula (35).


(35) $$r_{T}=\frac{h_{T-1}\left(s\right)\cdot f_{m}\left(f_{\mathrm{cnn}}\right)}{\left\|h_{T-1}\left(s\right)\cdot f_{m}\left(f_{\mathrm{cnn}}\right)\right\|}$$

According to formula (35), the total loss $r_{\text{loss}}$ is calculated in formula (36).


(36) $$r_{\text{loss}}=\beta \left(m_{\text{loss}}+r_{T}\right)$$

Where β is the hyperparameter with the range of (0,1).

## 4. Experimental process and result analysis

We assess the effectiveness of the image caption model presented in this paper by means of a deliberate experimental process, including thorough comparative analysis of the experimental results. The experimental environment and datasets deployed in the experiment are introduced in detail. Additionally, the data preprocessing method, specific model training methodology, and optimization of model parameters are also comprehensively discussed. Finally, through comparative analysis, the performance and advantages of the proposed model are evaluated in depth for maximum objectivity and credibility.

In the tasks of image caption, the most popular datasets adopted by most researchers include MS COCO (Lin et al., 2014) and Flickr 30 k (Young et al., 2014). The Flickr dataset is primarily a description of human activity scenarios. We use 29,000 of the Flickr data as a training set, 1,000 as a validation set, and the remaining 1,000 as a test set. In addition, 40,775 images and 30,775 data of the corresponding image descriptions from the MS COCO dataset are added to the training set to increase the number of training samples. The deep learning framework used is TensorFlow.

First of all, it is necessary to preprocess the data in the datasets, including the images and the descriptions. The image size is uniformly adjusted to 256*256, then trimmed to 224*224 to fit the model input. And the image is normalized to scale each pixel with the range of (0,1). Firstly, the description sentences need to segment, convert all letters to lower case, and remove spaces and punctuation. Then, the number of occurrences of all words in the datasets is counted, and words that appear less than 5 times are tagged *UNK* which have little effect on predicting outcomes. Finally, it is stipulated that the length of the sentences is not more than 15 words, each sentence only intercepts the characteristic values corresponding to the first 15 words. For sentences with less than 15 words, we supplement the number of characteristic values to 15, and the supplementary characteristic values are 0. At the same time, the tag *start* and *end, respectively,* placed at the beginning and end of the description sentences, to mark the beginning and end of the sentences.

In this paper, we adopt BLEU (Papineni et al., 2002), METEOR (Banerjee and Lavie, 2005), ROUGE-L (Lin, 2004), and CIDEr (Vedantam et al., 2015), which are commonly used evaluation indicators. In the model testing phase, this paper uses the method of beam search to choose a better generated sentence. The five sentences with the highest probability value are output at each decoding moment, that is, the value of beam size is set to 5.

**FIGURE 10:**
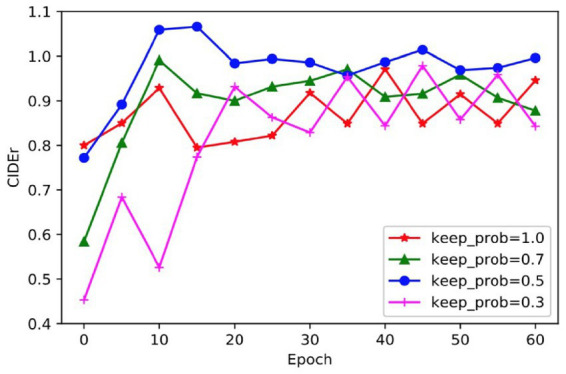
A comparison of the scores of different dropout ratio on CIDEr.

Given that dropout operation is used during model training, the impact of different dropout ratios on model performance can vary. To determine the optimal dropout ratio for the model, this paper compares model scores across different dropout ratios using the CIDEr evaluation indicator and presents a comparative graph in Figure 10. Analysis of the results indicates that when dropout operation is not performed, the score of the model fluctuates greatly, which indicates that the model is too complex and overfitting has occurred. Similarly, when the dropout ratio is 0.3, the fluctuation remains high and the model convergence score is low suggestive of underfitting arising from insufficient involvement of neurons in training. In contrast, when the dropout ratio is set at either 0.5 or 0.7, the curve remains relatively stable with a better CIDEr score when the dropout ratio is 0.5. Thus, the appropriate dropout ratio for the model is determined to be 0.5.

In this study, we conducted a comparative analysis of our model’s performance against other mainstream models, namely Google NIC, Soft attention, g-LSTM, RIC, RHN, and LSTM-A5. We evaluated the models using different metrics on MS COCO and Flickr 30 k. The comparison results are presented in Tables 1, 2.

**TABLE 1:** Comparison of model performance on MS COCO dataset.

Models	BLEU-1	BLEU-4	METEOR	ROUGE-L	CIDEr
Google NIC	0.666	0.277	0.237	-	0.855
Soft attention	0.707	0.243	0.239	-	-
g-LSTM	0.670	0.264	0.227	-	0.813
RIC	0.734	0.299	0.254	-	-
RHN	0.723	0.306	0.252	-	0.989
LSTM-A5	0.730	0.325	0.251	0.538	0.986
This paper (basic model with no DRL and attention mechanism)	0.716	0.289	0.244	0.456	0.893
This paper (final model with DRL but no attention mechanism)	0.746	0.339	0.284	0.583	0.991
This paper (final model with DRL and attention mechanism)	0.752	0.344	0.289	0.588	1.066

**TABLE 2:** Comparison of model performance on the Flickr 30 k dataset.

Models	BLEU-1	BLEU-4	METEOR	ROUGE-L	CIDEr
Google NIC	0.663	0.183	-	-	-
Soft attention	0.669	0.199	0.185	-	-
g-LSTM	0.646	0.206	0.180	-	-
RIC	0.745	0.244	0.202	-	-
RHN	0.738	0.307	0.216	-	-
This paper (basic model with no DRL and attention mechanism)	0.718	0.242	0.191	0.352	0.886
This paper (final model with DRL but no attention mechanism)	0.734	0.320	0.215	0.492	0.885
This paper (final model with DRL and attention mechanism)	0.738	0.335	0.222	0.504	0.921

As shown in Table 1, on the MS COCO dataset, the basic model proposed in this paper has improved the scores of BLEU-1 and BLEU-4, which measure sentence coherence and accuracy, by nearly 0.05 and 0.03, respectively, compared with the g-LSTM model, due to the use of the guided decoding network. At the same time, using DenseNet and MIL to process image information also improved the score of CIDEr evaluation index reflecting semantic richness by nearly 0.04 compared with Google NIC which only used the Inception-v3 structure as the image information extraction model. However, compared with more advanced models such as RIC and LSTM-A5, the proposed basic model still has a certain gap in the scores of various evaluation indexes. The reason is that the attention mechanism is not introduced, so the details are not enough. And the decoder only uses a single layer structure, so the decoding process is not sufficient.

As can be seen from the results in Table 1, on the MS COCO dataset, the performance of the final model in this paper is superior to the comparison models on various evaluation indicators even when without attention mechanism. Therefore, the use of DRL can significantly improve the performance of the image caption model, and when the attention mechanism is added, the model certainly performs better. Specifically, the BLEU scores of the proposed model are improved by 0.018 and 0.019, respectively, compared with the best results in the comparison models, which indicates that the output sentences of the proposed model have better coherence and accuracy. In terms of the METEOR scores, the proposed model also has an improvement of more than 0.03 compared with other models. In addition, without the attention mechanism, the model in this paper is also improved by more than 0.05 compared with the g-LSTM model, so the end-to-end model structure in this paper has greater advantages than the static adjustment of g-LSTM. Compared with the Soft attention model, which also uses the attention mechanism, the performance is improved by 0.05 due to the double-layer mechanism guiding the decoding and the optimization of DRL. In terms of CIDEr scores, which measures semantic richness and description consistency, there is also an improvement of 0.077 compared with the best results in the comparison models, which shows the excellent performance of the model designed in this paper.

As shown in Table 2, because the Flickr 30 k dataset contains much less data than the MS COCO dataset, the evaluation index scores of the proposed basic model and final model are basically decreased compared with those in Table 1. However, the basic model presented in this paper has higher evaluation index scores than the Google NIC, Soft attention, and g-LSTM models. And the scores of the final model are better than the comparison models in most evaluation indicators, however, the scores of some indicators are slightly lower than those of some models, which may be caused by the poor generalization ability of the model due to too small amount of data.

**FIGURE 11:**
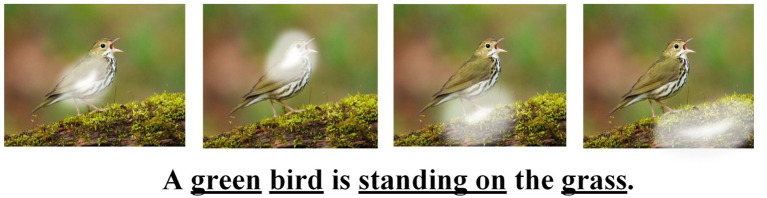
The effect diagram of the attention mechanism.

After the attention mechanism is used to improve the proposed model, in order to verify the actual effect, the extracted image features and the hidden layer state of the first layer decoder are processed by the attention module, then the words corresponding to different regions in the image are determined according to the corresponding weights, and the effect diagram is shown in Figure 11. Figure 11 shows the corresponding focus of each word in the sentence in the image. The white highlights in each image from left to right correspond to each word from left to right in the sentence below, and the whiter part of the highlights indicates the greater attention weight assigned. As can be seen from the images, the attribute word “green” about color focuses on the position of the bird’s body, and the target subject “bird” focuses on the head of the bird, because the head is the area that can best reflect the characteristics of the bird. The phrase “standing on” focuses on the bird’s feet, which is characteristic of the action. The word “grass” focuses on the green area where the bird is standing. Through the above analysis, it can be seen that the double-layer decoding structure model with the introduction of the attention mechanism is very accurate in extracting and matching key information and local information in the image, and it is also helpful in improving the performance of the image caption model.

## 5. Conclusion

Aiming at the problems of existing image caption models, this paper proposes an image caption model based on deep learning. Firstly, based on the NIC model, the encoder and decoder are optimized through DenseNet and NLSTM networks. Meanwhile, this paper also introduces a guided decoding network to realize the dynamic adjustment of encoded information in the decoding process and avoid the loss of image information. The experimental results show that compared with several common models, the performance of the basic model designed in this paper is improved. Then, on the basis of the proposed image caption model, we introduce the attention mechanism to construct a double-layer decoding structure and improve the decoding depth to obtain the details of the image. The powerful perception and decision abilities of DRL are adopted to optimize the model, which solve the problem of discrepancies between training objectives and evaluation indicators, and improve the expressive ability of the image caption model. Through the comparison and analysis of the experimental results with several common models, our image caption model further improves the scores of each evaluation index, and the output description of the image is more accurate and semantic rich. In future work, we will design the image caption model based on expression ways in different scenes and language habits of different people, so that the sentences output by the model will be closer to the expression ways of humans in real scenes. Meanwhile, we will continue to expand the datasets to include richer content, and further design a better model to enable zero-sample learning through textual inference.

## Data availability statement

The original contributions presented in the study are included in the article/supplementary material, further inquiries can be directed to the corresponding author.

## Author contributions

TB: Methodology, Project administration, Writing – original draft, Writing – review & editing. SZ: Software, Supervision, Validation, Writing – review & editing. YP: Data curation, Supervision, Writing – review & editing. JL: Validation, Visualization, Writing – original draft. HW: Data curation, Writing – original draft, Writing – review & editing. YD: Investigation, Writing – original draft.
